# Correction: eIF4E S209 phosphorylation licenses myc- and stress-driven oncogenesis

**DOI:** 10.7554/eLife.109900

**Published:** 2025-11-14

**Authors:** Hang Ruan, Xiangyun Li, Xiang Xu, Brian J Leibowitz, Jingshan Tong, Lujia Chen, Luoquan Ao, Wei Xing, Jianhua Luo, Yanping Yu, Robert E Schoen, Nahum Sonenberg, Xinghua Lu, Lin Zhang, Jian Yu

**Keywords:** Human, Mouse

Ruan H, Li X, Xu X, Leibowitz BJ, Tong J, Chen L, Ao L, Xing W, Luo J, Yu Y, Schoen RE, Sonenberg N, Lu X, Zhang L, Yu J. 2020. eIF4E S209 phosphorylation licenses myc- and stress-driven oncogenesis. *eLife* **9**:e60151. doi: 10.7554/eLife.60151.

Published 2 November 2020

We were notified via PubPeer of two errors. In Figure 5A, the WT control (GLU +) well was inadvertently duplicated in KI control (GLN +). In Figure 7-S1, the GCN2 blot of RKO cells was inadvertently duplicated in that of HT 29 cells on the right. We predicted that these duplications occurred during figure preparation and composite using different software. Due to high similarity in these images, the authors failed to recognize them even after repeated proofreading.

We have since retrieved full plate and gel scan images and replaced the duplication with correct images. The authors also noted that the link to deposited microarray data in Material of Method was incorrect while that in Data availability statement is correct. These corrections do not change the results or conclusions of our study.

Corrected text under Data Deposit: Microarray data have been deposited at DRYAD (https://dx.doi.org/10.5061/dryad.tb2rbnzxm).

Original text under Data Deposit: Microarray data have been deposited at DRYAD (https://orcid.org/0000-0002-4021-1000) and will be released to the public upon publication of manuscript.

The corrected Figure 5 (updated for panel A, KI GLN+) is shown here:

**Figure 1:**
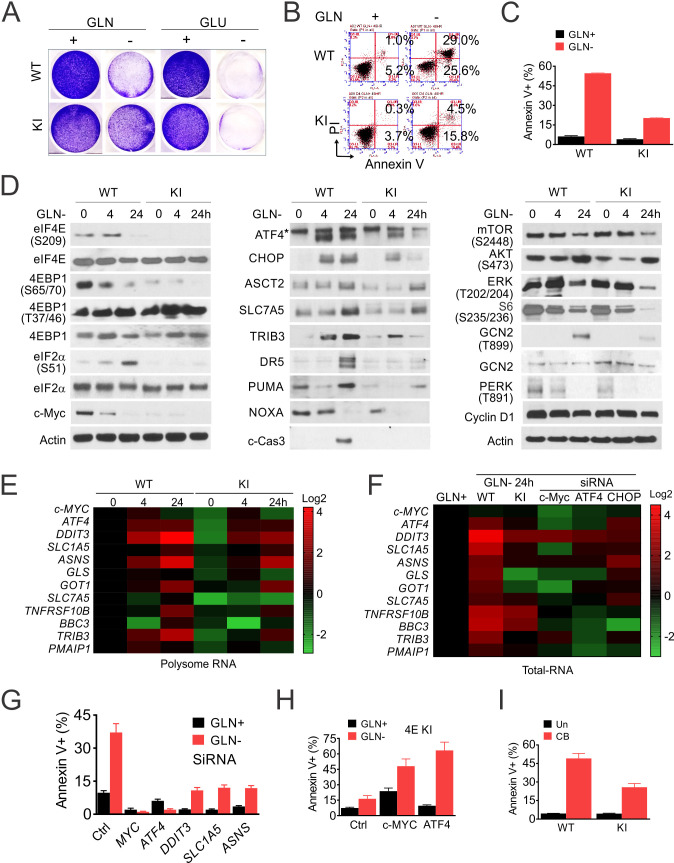


The originally published Figure 5 is shown for reference:

**Figure 2:**
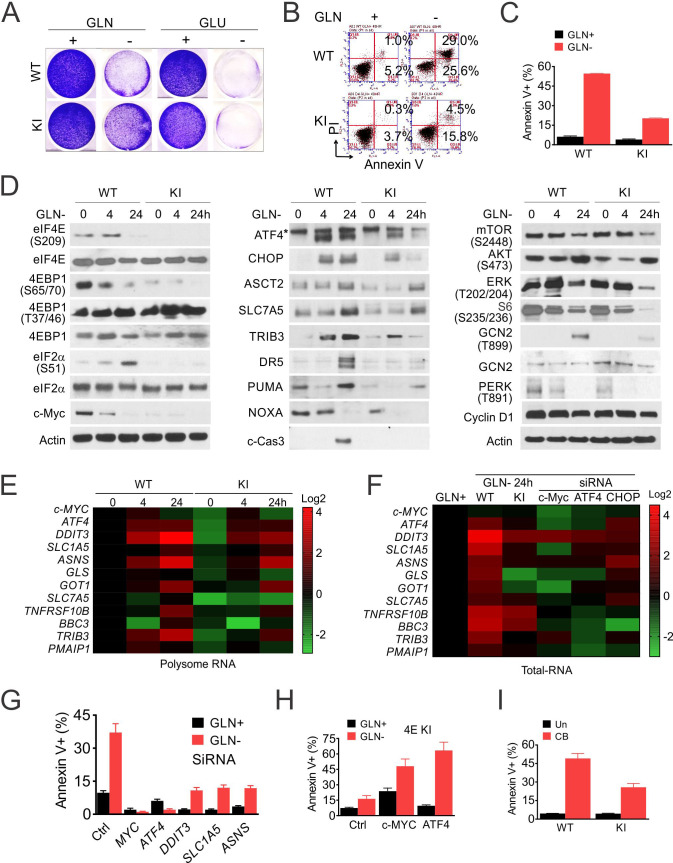


The corrected Figure 7—figure supplement 1 (updated for the HT29 p-GCN panel) is shown here:

**Figure 3:**
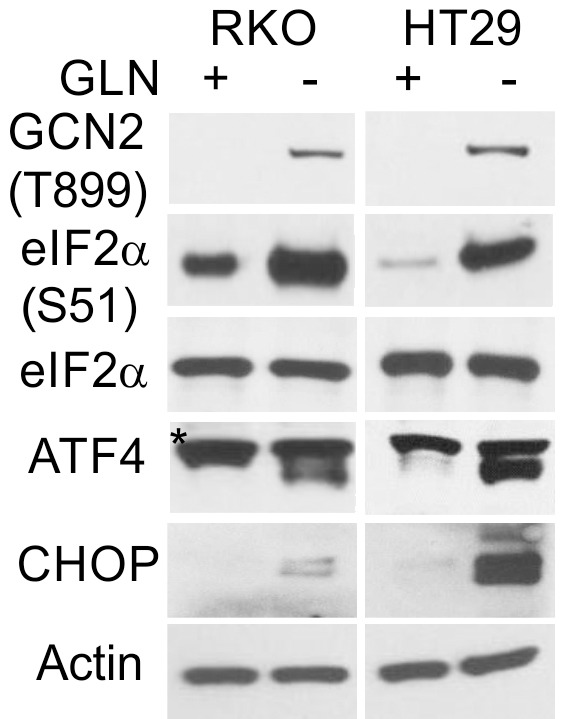


The originally published Figure 7—figure supplement 1 is shown for reference:

**Figure 4:**
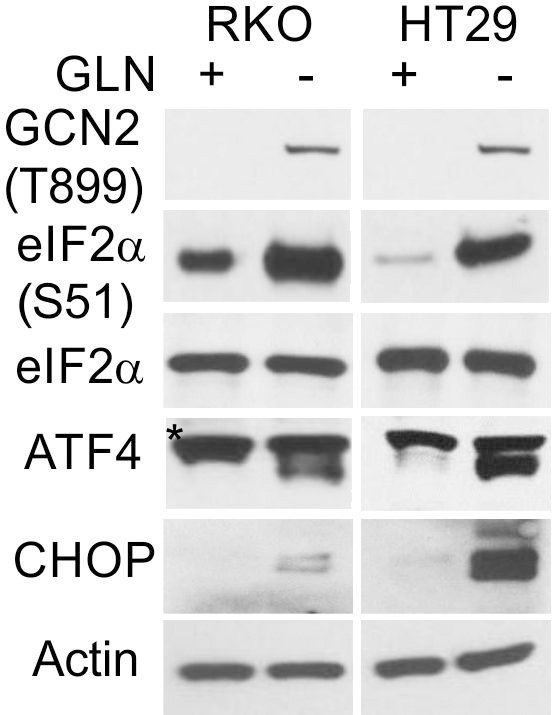


The article has been corrected accordingly.

