# Supplementary material for: eIF4E S209 phosphorylation licenses myc- and stress-driven oncogenesis
Source: eLife. 2020 Nov 2;9:e60151. doi: 10.7554/eLife.60151 (PMC7665890; doi:10.7554/eLife.60151)
Supplement: Supplementary file 1. [file elife-60151-supp1.docx]

**Supplementary file 1. Differential and selected genes in WT and 4EKI cells**

| **1A. Top down-regulated genes in 4EKI cells.** | | | | | |
| --- | --- | --- | --- | --- | --- |
| **Gene** | **Function** | **WT signal** | **KI signal** | **fold** | **p value** |
|  |  |  |  |  |  |
| **DDIT3 (CHOP)** | ER or other stress | 3944 | 389.4 | 10.1 | 2E-05 |
| **GADD45B** | ER or other stress | 405.5 | 50 | 8.1 | 0.000214 |
| **ATF3** | ER or other stress, TF | 3198.3 | 785.8 | 4.1 | 3E-05 |
| **GADD34** | ER or other stress | 1223.9 | 300.8 | 4.1 | 0.000774 |
| **GADD45A** | ER or other stress | 6648.9 | 2872.4 | 2.3 | 2E-05 |
| **GRP78** | ER or other stress | 28832.1 | 14127 | 2.0 | 4E-05 |
| **ATF4** | ER or other stress, TF | 18797.8 | 9634.7 | 2.0 | 2E-05 |
|  |  |  |  |  |  |
| **ASNS** | metabolism, Gln | 27072.8 | 1829.1 | 14.8 | 2E-05 |
| **SLC7A11 (xCT)** | metabolism, Gln | 6958.8 | 1182 | 5.9 | 2E-05 |
| **GOT 1** | metabolism, Gln | 9365.9 | 1794.7 | 5.2 | 2.3E-05 |
| **SLC3A2 (CD98)** | AA transporter | 6104.9 | 1213.4 | 5.0 | 2E-05 |
| **SLC7A5 (LAT1)** | AA transporter | 9848.3 | 2870.3 | 3.4 | 2E-05 |
| **SLC38A1 (ATA1)** | AA transporter | 2964.1 | 1033.7 | 2.9 | 2E-05 |
|  |  |  |  |  |  |
| **WARS** | translation | 4875.4 | 970.4 | 5.0 | 2E-05 |
| **CARS** | translation | 13895.8 | 3576.6 | 3.9 | 2E-05 |
| **YARS** | translation | 11921.1 | 3653.6 | 3.3 | 2E-05 |
| **GARS** | translation | 15060.1 | 5270 | 2.9 | 2E-05 |
| **MARS** | translation | 3911.6 | 1393.3 | 2.8 | 2E-05 |

| **1B. Expression of other Myc and ISR targets in WT and KI cells** | | | | | |
| --- | --- | --- | --- | --- | --- |
| **Gene** | **Function** | **WT signal** | **KI signal** | **fold** | **p value** |
| **EIF4E** | translation | 867.4 | 666.9 | 1.3 | 0.138386 |
| **LDHA** | glycolysis | 22945.7 | 32609.9 | 0.7 | 0.9998 |
| **SLC2A1(GLUT)** | glycolysis | 2740 | 2229 | 1.2 | 0.442 |
| **CAD** | nucleotide synthesis | 2827.4 | 2640.1 | 1.1 | 0.805199 |
| **PPAT** | nucleotide synthesis | 3181 | 2193 | 1.5 | 0.000241 |
| **SHMT2** | nucleotide synthesis | 2341.6 | 1708.5 | 1.4 | 0.001832 |
| **FASN** | lipid metabolism | 9.2 | 14.2 | 0.7 | 0.894337 |
| **SCD (FADS3)** | lipid metabolism | 11807.3 | 10428.8 | 1.1 | 0.5 |
| **TRIB3** | cell death | 2736.7 | 300.4 | 9.11019 | 0.00013 |
| TNFRSF10B | cell death | 10774.6 | 3911.3 | 2.8 | 2.3E-05 |
| **BBC3 (PUMA)** | cell death | 843 | 688.2 | 1.22493 | 0.2 |
| **PMAIP1 (NOXA)** | cell death | 2551.3 | 1839.4 | 1.38703 | 0.000244 |
