# Supplementary material for: eIF4E S209 phosphorylation licenses myc- and stress-driven oncogenesis
Source: eLife. 2020 Nov 2;9:e60151. doi: 10.7554/eLife.60151 (PMC7665890; doi:10.7554/eLife.60151)
Supplement: Supplementary file 2. [file elife-60151-supp2.docx]

**Supplementary file 2. Driver information in cell lines used**

| **Driver information in cell lines used** | | | | | | | | |
| --- | --- | --- | --- | --- | --- | --- | --- | --- |
| **Cell Line** | **KRAS** | **BRAF** | **PIK3CA** | **PTEN** | **TP53** | **APC** | **CTNNB1** | **MSI** |
| **HCT116** | G13D/+ | WT | H1047R | WT | WT | WT | S45del | MSI-H |
| **HCT116 (KRAS WT)** | WT (+/-) | WT | H1047R | WT | WT | WT | S45del | MSI-H |
| **HCT116 G13D** | G13D/- | WT | H1047R | WT | WT | WT | S45del | MSI-H |
| **SW480** | G12V | WT | WT | WT | R273H, P309S | Q1338* | WT | MSS |
| **DLD-1** | G13D | WT | E545K, D549N | WT | S241F | R727M | WT | MSI-H |
| **HT-29** | WT | V600E | P449T | WT | R273H | E853* , E1554fs | WT | MSS |
| **RKO** | WT | V600E | H1047R | WT | WT | WT | WT | MSI-H |
| **SW48** | WT | WT | G914R | WT | WT | R2714C | S33Y | MSI-H |
| **SW48/G13D** | +/G13D | WT | G914R | WT | WT | R2714C | S33Y | MSI-H |
| **SW48/G12V** | +/G12V | WT | G914R | WT | WT | R2714C | S33Y | MSI-H |
| **Lim1215** | WT | WT | WT | WT | WT | WT | T41A,Q177P | MSI-H |
| **Lim1215/G13D** | +/G13D | WT | WT | WT | WT | WT | T41A,Q177P | MSI-H |
| **Lim1215/G12V** | +/G12V | WT | WT | WT | WT | WT | T41A,Q177P | MSI-H |
