# Supplementary material for: eIF4E S209 phosphorylation licenses myc- and stress-driven oncogenesis
Source: eLife. 2020 Nov 2;9:e60151. doi: 10.7554/eLife.60151 (PMC7665890; doi:10.7554/eLife.60151)
Supplement: Supplementary file 3. [file elife-60151-supp3.docx]

**Supplementary file 3. Key reagents used in study**

| **3A. 4EKI primers (human and mouse)** | | |
| --- | --- | --- |
| **Gene** | **direction** | **Sequence (5'-3')** |
| 4EKI Lharm-F | Forward | GGG AAA GUg ggg gaa ggt agt aaa tct tct gg- 3’ |
| 4EKI Lharm-R | Reverse | GGA GAC AUt gaa aca ctt cga ggg aga atg t -3’ |
| 4EKI Rharm-F | Forward | GGT CCC AUa att gtg ctg aaa gga aaa ggg tg- 3’ |
| 4EKI Rharm-R | Reverse | GGC ATA GUt att cct gga tcc ttc acc aat gt- 3’ |
| LH screening-F | Forward | cat ttc atg cta tat ggg gtt cca- 3’ |
| NeoR | Reverse | ttg tgc cca gtc ata gcc g-3’ |
| NeoF | Forward | tct tga cga gtt ctt ctt ag-3’ |
| RH screening-R | Reverse | cta gtg ctc caa act tat gct gt- 3’ |
|  |  |  |
| NeoF | Forward | tct tga cga gtt ctt ctt ag-3’ |
| RH 2nd round screening | Forward | aca gta tca cag ccc tta tg- 3’ |
| RH 2nd round screening | reserve | gtg gag ccg ctc tta gta g- 3’ |
|  |  |  |
| m4EKI | Forward | TTTGAAATTGGTTTGTAAAGTTGG |
| m4EKI | Reverse | GCAATGCAAGTCGAAATGTG |
| Apc/Min | oIMR0033 | GCCATCCCTTCACGTTAG |
| Apc/Min | oIMR0034 | TTCCACTTTGGCATAAGGC |
| Apc/Min | oIMR0758 | TTCTGAGAAAGACAGAAGTTA |

| **3B. qRT-PCR primers used (human)** | | |
| --- | --- | --- |
| **Gene** | **direction** | **Sequence (5'-3')** |
| ACTIN | Forward | GACATTAAGGAGAAGCTGTGCTATGTT |
| ACTIN | Reverse | GCCTAGAAGCATTTGCGGTGGACGA |
| SLC1A5 (ASCT2) | Forward | TCCAGCCCTCGGGAGTAAAT |
| SLC1A5 (ASCT2) | Reverse | CGGATAAGCAGCTCCCCTTC |
| ASNS | Forward | GGGGCTTGGACTCCAGCTTG |
| ASNS | Reverse | GAGCCTGAATGCCTTCCTCA |
| ATF4 | Forward | GTTCTCCAGCGACAAGGCTA |
| ATF4 | Reverse | GTGTCATCCAACGTGGTCAG |
| CARS | Forward | CAGCCATGGCAGATTCCT |
| CARS | Reverse | AGGCTGTTGTAAAGGTGGAGTC |
| DDIT3 (CHOP) | Forward | TGGAAATGAAGAGGAAGAATCAAAA |
| DDIT3 (CHOP) | Reverse | CTTGGTGCAGATTCACCATTC |
| c-MYC | Forward | TCTTGACATTCTCCTCGGTGTCCGAGGACCT |
| c-MYC | Reverse | TACCCTCTCAACGACAGCAGCTCGCCCAACTCCT |
| CCND1 | Forward | GGGCAGCAGAAGCGAGAG |
| CCND1 | Reverse | GTTCCTCGCAGACCTCCAG |
| TNFRSF10B (DR5) | Forward | AAGACCCTTGTGCTCGTTGT |
| TNFRSF10B (DR5) | Reverse | AGGTGGACACAATCCCTCTG |
| EIF4E | Forward | TTGAGAACCGCGCACCCTA |
| EIF4E | Reverse | GTGGTTTCCGGTTCGACAGT |
| FASN | Forward | ACCAGAGCAGCCATGGAG |
| FASN | Reverse | TTGATGCCTCCGTCCACGAT |
| FBXO | Forward | CTCAGTGGATGTTTGAACTTGCT |
| FBXO | Reverse | CCTTTGGTATCTGCCGATGTTTT |
| GARS | Forward | ATGGAGGTGTTAGTGGTCTGT |
| GARS | Reverse | CTGTTCCTCTTGGATAAAGTGCT |
| GLS | Forward | GCAGTTTGCTTTCCATGTTGG |
| GLS | Reverse | AGCAAGTTCTTGTTGGAGACT |
| GOT1 | Forward | ATGGCACCTCCGTCAGTCT |
| GOT1 | Reverse | AGTCATCCGTGCGATATGCTC |
| LDHA | Forward | GACGTGCATTCCCGATTCCT |
| LDHA | Reverse | AGATATCCACTTTGCCAGAGACA |
| MARS | Forward | AGGCCCGGAAGATTGTGTG |
| MARS | Reverse | AGAGGTAGTTGCCGCTATCCA |
| MMP7 | Forward | GAGTGAGCTACAGTGGGAACA |
| MMP7 | Reverse | CTATGACGCGGGAGTTTAACAT |
| PGC1B | Forward | ACTATCTCGCTGACACGCAG |
| PGC1B | Reverse | TGTCAATCTGGAAGAGCTCGG |
| PPAT | Forward | AGGTCCTTCCACGTGCTTTC |
| PPAT | Reverse | ACAAGACCCATTCCCTTGTGT |
| BBC3 (PUMA) | Forward | CGACCTCAACGCACAGTACGA |
| BBC3 (PUMA) | Reverse | AGGCACCTAATTGGGCTCCAT |
| RPL21 | Forward | CAAGGGAATGGGTACTGTTCAAA |
| RPL21 | Reverse | CTCGGCTCTTAGAGTGCTTAATG |
| SARS | Forward | CAGCCCTCATCCGAGAGAC |
| SARS | Reverse | TCTGCCCGAAATCTACATCGT |
| SLC7A11 | Forward | TCTCCAAAGGAGGTTACCTGC |
| SLC7A11 | Reverse | AGACTCCCCTCAGTAAAGTGAC |
| SLC7A5 | Forward | TTGACACCACTAAGATGAT |
| SLC7A5 | Reverse | GTAGCAATGAGGTTCCAA |
| TFAM | Forward | TCCCATAGTGCCTCGCTAGT |
| TFAM | Reverse | CACAAAACTGAAGGGGGAGC |
| TRIB3 | Forward | TGGTACCCAGCTCCTCTACG |
| TRIB3 | Reverse | GACAAAGCGACACAGCTTGA |
| WARS | Forward | CAACCCACCCCTGATTGGAC |
| WARS | Reverse | CAGACGAGTCAAGACCAGGTT |
| YARS | Forward | TGGTCACACAGCACGATTCC |
| YARS | Reverse | CGGGGTATAAGAGGCCACTC |

| **3C. qRT-PCR primers used (mouse)** | | |
| --- | --- | --- |
| **Gene** | **Primer** | **Sequence (5'-3')** |
| Eif4e | Forward | GGACCAGGAATGTGCTTCAT |
| Eif4e | Reverse | CCCAACACTACACCTCATCTTC |
| Myc | Forward | TCTCCACTCACCAGCACAACTACG |
| Myc | Reverse | ATCTGCTTCAGGACCCT |
| Atf4 | Forward | GAGCTTCCTGAACAGCGAAGTG |
| Atf4 | Reverse | TGGCCACCTCCAGATAGTCATC |
| Ddit3 (Chop) | Forward | CTGCCTTTCACCTTGGAGAC |
| Ddit3 (Chop) | Reverse | CGTTTCCTGGGGATGAGATA |
| Slc1a4 (Asct1) | Forward | TGCTCTGGCGTTCATCATCA |
| Slc1a4 (Asct1) | Reverse | AGTGAATGCGGCAACCACAA |
| Slc1a5 (Asct2) | Forward | TCAACCATGGTCCAGCTTCT |
| Slc1a5 (Asct2) | Reverse | CGGGTGCGTACCACATAATC |
| Gls | Forward | CCATCAAGCCTCACATCTCTAC |
| Gls | Reverse | GTGCAGTTTCGCTGTCTTTAC |
| Got1 | Forward | CCCAAGCAGGTCGAGTATTT |
| Got1 | Reverse | TGGAGGTAGCGACGTAATCTA |
| Mars | Forward | GCAAGGTATTGTCGCCTTCG |
| Mars | Reverse | CCAGCGGTAGATGTCAGCAT |
| Gars | Forward | GGAAGGCGCTATGCAAGAAC |
| Gars | Reverse | GAGTCTCGGTCCCTCAGAGT |
| Yars | Forward | CCCAGATGCAGATAGCCTTTAT |
| Yars | Reverse | CCTGTCCTGCAGTTCTTCTT |
| Rpl21 | Forward | AGCACACTTTGTGAGGACTAAT |
| Rpl21 | Reverse | CAGTCCTGGTGCTGGTATTT |
| Tnfrsf10b (Dr5) | Forward | AAAACGGCTTGGGCATCTTGGC |
| Tnfrsf10b (Dr5) | Reverse | AGACGGTTCCAGGAGTCAAAGG |
| Bbc3 (Puma) | Forward | ATGGCGGACGACCTCAAC |
| Bbc3 (Puma) | Reverse | AGTCCCATGAAGAGATTGTACATGAC |
| Trib3 | Forward | TGGCTGGCAGATACCCATTC |
| Trib3 | Reverse | CAAGTCGCTCTGAAGGTTCCTT |
| Pmaip1(Noxa) | Forward | CCCAGATTGGGGACCTTAGT |
| Pmaip1(Noxa) | Reverse | CTGCGAACTCAGGTGGTAGC |
| Gapdh | Forward | CTCTGGAAAGCTGTGGCGTGATG |
| Gapdh | Reverse | ATGCCAGTGAGCTTCCCGTTCAG |

| **3D. Antibodies used (human and mouse)** | | | | | |
| --- | --- | --- | --- | --- | --- |
| **ANTIGEN** | **VENDOR** | **CATALOG** | **APPLICATION** | **DILUTION** | **H or M** |
| 4E-BP1 | Cell Signaling | #9452 | IB, PLA | 1/1000, 1/100 | h |
| 4E-BP1(S65/70) | Cell Signaling | #9451 | IB, IF | 1/1000,1/100 | h, m |
| 4E-BP1(37/46) | Cell Signaling | #2855 | IB | 1/1000 | h |
| eIF4E | Santa Cruz | SC-9976 | IB, PLA | 1/1000, 1/100 | h, m |
| eIF4E (S209) | Cell Signaling | #9741S | IB, IF, IHC | 1/1000,1/100,1/100 | h |
| eIF4E (S209) | Abcam | 76256 | IF | 1:100 | m |
| eIF4G1 | bethyl | A300-502A | IB | 1/1000 | h |
| eIF2-alpha | Santa Cruz | SC-11386 | IB | 1/1000 | h, m |
| eIF2 alpha (S51) | Cell Signaling | #3398 | IB, IF | 1/1000,1/100 | h, m |
| Actin (Beta) | Sigma | A5441 | IB | 1/1000 | h, m |
| AKT(S473) | Cell Signaling | #9271 | IB, IF | 1/1000,1/100 | h, m |
| ATF4 (CREB-2) | Santa Cruz | SC-200 | IB | 1/1000 | h |
| Bcl-xL | Cell Signaling | #2764 | IB | 1/1000 | h |
| CHOP (L63F7) | Cell Signaling | #2895s | IB | 1/1000 | h |
| Caspase-3, cleaved | Cell Signaling | #9661 | IB | 1/1000 | h, m |
| c-Myc | Santa Cruz | SC-40 (9E10) | IB | 1/500 | h |
| c-Myc | Abcam | 39688 | IF | 1/100 | h, m |
| Cyclin D1 | Santa Cruz | SC-8396 | IB | 1/1000 | h |
| ERK (T202/204) | Cell Signaling | #9101 | IB | 1/500 | h |
| Flag (M2 Ab) | Sigma | F3165 | IB | 1/1000 | N/A |
| GCN2 | Abcam | Ab137543 | IB | 1/1000 | h |
| GCN2(T899) | Abcam | Ab75836 | IB, IF | 1/500,1/100 | h |
| HA-probe(Y-11) | Santa Cruz | SC805 | IB | 1/1000 | N/A |
| Ki67 | DAKO | M7249 | IF, IHC | 1/100,1/100 | m |
| mTOR (S2448) | Cell Signaling | #5536 | IB | 1/1000 | h, m |
| MMP7 | EMD Biosciences | IM40 | IB | 1/1000 | h |
| PERK (T981) | Santa Cruz | SC-32577 | IB | 1/1000 | h |
| S6 (235/236) | Cell Signaling | #2211 | IB, IF | 1/1000,1/100 | h, m |

| **3E. siRNA used (Human-specific)** | |
| --- | --- |
| **Gene** | **Sequence** |
| c-MYC | 5′-AACGUUAGCUUCACCAACAUU-3’ |
| ATF4 | 5’-GCCUAGGUCUCUUAGAUGA(dTdT)-3’ |
| DDIT3 | 5’-GCACAGCTAGCTGAAGAGA (dTdT)-3’ |
| SLC1A5 | 5’-GCUCAUACUCUACCACCUA(dTdT)-3’ |
| ASNS | 5’-GCAUCCGUGGAAAUGGUUA(dTdT)-3’ |
| Scramble | 5’-AACGTACGCGGAATACTTCGA dTdT)-3’ |

| **3F. Constructs and other key reagents used** | | | |
| --- | --- | --- | --- |
| **Name** | **Vendor** | **Catalog No.** | **Application** |
| cDNA-LUC | N/A | N/A | Reporter |
| HA-ECFP | N/A | N/A | Reporter |
| c-Myc-EYFP | N/A | N/A | Reporter |
| pRTK-ATF4 | Addgene | #26114 | Expression |
| pcDNA3.3 c-MYC | Addgene | #26818 | Expression |
| m7GTP | Jena Bioscience | AC-155L | pull down |
| Duolink In Situ Redstarter Kit | Sigma | DUO92101-1KT | 4E/4EBP1 PLA |
| DMEM (Gln and Pyruvate free) | Giboco | 15-017-CV | culture |
| dialyzed FBS | Giboco | A3382001 | culture |
| glutamine | Corning Cellgro | #25-005-CI | culture |
| sodium pyruvate | Giboco | #11360070 | culture |
| C-B839 | MedChemExpress | HY-12248 | GLS inhibitor |
| Eft508 | Selleckchem | #S8275 | Mnk inhibitor |
| advanced DMEM/F12 | Invitrogen | 12634-010 | organoids |
| penicillin/streptomycin | Invitrogen | 15140-122 | organoids |
| GlutaMAX | Invitrogen | 35050-061 | organoids |
| HEPES | Invitrogen | 15630-106 | organoids |
| B27 | Invitrogen | 17504-044 | organoids |
| N2 | Invitrogen | 17502-048 | organoids |
| N-Acetylcysteine | Sigma | A0737 | organoids |
| [leu-15]-Gastrin | Sigma | G9145 | organoids |
| nicotinamide | Sigma | N0636 | organoids |
| SB202190 | Sigma | S7067 | organoids |
| A83-01 | Tocris Bioscience | 2939 | organoids |
| Y-27632 | Sigma | Y0503 | organoids |
| Primocin | ant-pm-1 | InvivoGen | organoids |
